# Predicting factors of blood pressure normalization in hypertensive patients after short-term follow-up

**DOI:** 10.3389/fcvm.2024.1403214

**Published:** 2024-08-27

**Authors:** Fatouma Sall, Gueu Christophe Meneas, Balayssac Ahou Edwige Siransy, Marie-Paule N’cho Mottoh, Yannik-Hermann Kpi, Ismael N’guessan, Vierge Marie Assi, Florent Diby, Anicet Adoubi

**Affiliations:** ^1^ Cardiology Department, Bouake Teaching Hospital, Alassane Ouattara University, Bouake, Cote d'Ivoire; ^2^ Cardiology Department, Institute of Cardiology of Abidjan, Felix Houphouet-Boigny University, Abidjan, Cote d'Ivoire

**Keywords:** normalization, arterial hypertension, cardiology, predicting factors, follow-up

## Abstract

**Introduction:**

Normalization of blood pressure in hypertensive patients is a major challenge for practitioners. Knowledge of the factors associated with normalization of blood pressure could help optimize management of these hypertensive patients. In this study, we analysed the factors predictive of this in a population of hypertensive patients followed as outpatients in a specialised department.

**Patients and methods:**

Retrospective and analytic study (January 2021–May 2022) of adult hypertensive patients over 40 years old who had been receiving antihypertensive treatment as outpatients in the Cardiology Department of the Bouake Teaching Hospital for at least 6 months. We studied the epidemiological and clinical parameters as well as the factors involved in the normalization of blood pressure in this population. Statistical analysis was performed using SPPS version 26 software (SPSS Inc., Chicago, IL, USA).

**Results:**

We collected 194 patients records (57.7% women). The mean age was 59.13 years [extremes: 40–89 years]. One hundred and nine (56.2%) patients had a low socioeconomic status and 151 (77.83%) had at least 2 cardiovascular risk factors. The mean systolic blood pressure on admission was 171.12 ± 22.38 mmHg [extremes: 140–259 mmHg] and the mean diastolic blood pressure was 97.98 ± 17.83 mmHg [extremes: 60–168 mmHg]. First-line treatment consisted of dual anti-hypertensive therapy (*n* = 133; 68.55%) and fixed combination (*n* = 152; 78.35%). Only 25.25% (*n* = 49) of patients achieved normalization of blood pressure with therapeutic adherence estimated at 37.62% (*n* = 73). In multivariate analysis adjusted for anti-hypertensive treatment adherence, age (OR = 1.03; 95% CI = 1.002–1.059; *p* = 0.039), absence of alcoholism (OR = 9.48; 95% CI = 2.13–42.11; *p* = 0.003), number of cardiovascular risk factors <2 (OR = 1.52; 95% CI = 1.06–2.16; *p* = 0.021), normalization of uricemia (OR = 1.05; 95% CI = 1.00–1.11; *p* = 0.039) and natraemia (OR = 1.01; 95% CI = 1.00–1.03; *p* = 0.021), dual therapy (OR = 0.40; 95% CI = 0.18–0.90; *p* = 0.027), change in treatment for optimization (OR = 4.22; 95% CI = 1.71–10.37; *p* = 0.002), intellectual education (OR = 10.40; 95% CI = 4.31–25.10; *p* < 0.001) and health insurance (OR = 0.09; 95% CI = 0.04–0.21; *p* < 0.001) were the main factors predicting normalization of blood pressure.

**Conclusion:**

Control of cardiovascular risk factors and compliance with treatment are the main factors in normalizing blood pressure.

## Introduction

Worldwide, approximately 1.39 billion people have hypertension (1). Hypertension is a major public health problem and one of the main modifiable risk factors for cardiovascular disease (1–3). In recent decades, in the era of epidemiological transition, we have witnessed an upsurge in this global scourge in our developing countries, with increased morbidity and mortality (4, 5). The annual mortality rate from hypertension worldwide is estimated at around 10.4 million people (6, 7). This mortality is partly due to uncontrolled high blood pressure levels, which are responsible for multiple complications such as stroke, ischaemic heart disease, congestive heart failure, kidney disease and hypertensive emergencies (6, 8, 9). According to learned societies, normalized blood pressure (BP) is only achieved when the target BP of less than 130/80 mmHg is reached (10–13). In current medical practice, this target is difficult to achieve and blood pressure in general is not optimally controlled (4, 14). Only 1/3 of patients on pharmacological treatment achieve the recommended blood pressure targets (4). In addition, it has been shown that a prolonged and effective reduction in blood pressure (BP) of 2 mmHg in hypertensive patients significantly reduces the risk of cardiovascular events by up to 10%, and a reduction in systolic blood pressure of 20 mmHg reduces coronary heart disease and stroke mortality by 50% (15). Thus, normalizing blood pressure levels in hypertensive patients should be a major challenge for practitioners (1), and identifying the factors associated with normalization could help optimize the management of hypertension. In this study, we analysed the predictive factors of normalization of blood pressure in a population of hypertensive patients followed up on an outpatient basis.

## Patients and methods

This was a retrospective and transversal study conducted over a 15-month period from January 01, 2021 to May 31, 2022. We included consecutively, adult hypertensive patients over 40 years of age, followed up on a short-term basis and receiving antihypertensive treatment on an outpatient basis in the cardiology department of Bouake University Hospital. These patients had undergone 3 successive consultations: (1) Initial consultation (M0), (2) Consultation at 3rd month (M3) and (3) Consultation at 6th month (M6). Short-term follow-up is defined as follow-up during the first year outside the 3-month initiation phase, in accordance with the ESH international guidelines (16). Patients with secondary arterial hypertension, hypertensive pregnant women and pregnant arterial hypertension were not included. Hypertension was defined as a BP ≥140/90 mmHg. For Patients aged 18–64 years old and 65–79 years old, the office Blood pressure (BP) was considered normalized when it's <130/80 mmHg and <140/80 mmHg respectively. For Patients aged ≥80 years old, the office Systolic Blood pressure (SBP) was considered normalized when it's in the 140–150 mmHg range (16). Blood pressure was measured using an OMRON M6 automatic electronic blood pressure monitor (15). Measurements were taken in the sitting position, using a cuff adapted to the patients' morphology. An average of 02 measurements was considered for analysis (17). The body mass index used as an index of obesity was calculated from the weight in kilograms divided by the square of the height in meters. Secondary education or above was considered intellectual education. Anyone living on less than $2.15 a day was considered in low socioeconomic status (18). We analyzed the epidemiological and clinical parameters and the factors influencing the normalization of blood pressure. To evaluate the therapeutic adherence, we used the indirect method (Self report) based on patient interview (19). Our therapeutic strategy was based on the decision-making algorithm recommended by the 2018 ESH guidelines (12). The data were collected on an individual survey form, filled in according to the various parameters studied, from the medical records. Statistical analysis was performed using Epi info 7 software and SPPS version 26 (SPSS Inc., Chicago, IL, USA). Qualitative values were expressed as percentages and quantitative variables as mean ± standard deviation. We used the Kolmogorov–Smirnov to assess the normally distribution of the different continous variables. Comparisons were made using ANOVA for quantitative variables and the Chi^2^ test for qualitative variables. The multivariate analysis consisted of a logistic regression analysis with adjustment of the variables studied for therapeutic adherence. A *p*-value < 0.05 was considered statistically significant.

## Results

### Epidemiological and clinical aspects

We collected 194 records from adult hypertensive patients. The mean age was 59.13 years [extremes: 40–89 years] and 57.7% were women. Epidemiological and clinical characteristics are listed in Table 1. One hundred and nine (56.18%) patients were in low socioeconomic status and 151 (77.83%) had at least 2 cardiovascular risk factors. Dyslipidaemia (26.8%), obesity (17.52%), diabetes (10.30%) and smoking (7.73%) were the most common cardiovascular risk factors in addition to hypertension. Blood pressure on admission averaged 171.12 (140–259) mmHg for systolic blood pressure and 97.46 (60–168) mmHg for diastolic blood pressure. The high blood pressure symptoms were present in 44.84%, including the headache (23.19%), vertigo (9.79%), tinnitus (6.70%) and phosphenes (5.15%).

**Table 1:** Epidemiological and clinical characteristics.

Variables	*N* = 194	(%)
Age, mean (years) (extremes)	59.13 (40–89)	–
Female gender	112	57.7
Low socio-economic level	109	56.2
Diabetes	20	10.30
Type 1 diabetes	6	3.1
Type 2 diabetes	14	7.2
Smoking	15	7.7
Dyslipidaemia	52	26.8
Obesity	34	15.5
Heredity	16	8.24
Alcoholism	9	4.6
Stress	4	2.06
Chronic renal failure	2	1
<2 CVRF	43	22.16
≥2 CVRF	151	77.83
Systolic blood pressure (mmHg), mean ± SD (extremes)	171.12 ± 22.38 (140–259)	–
Diastolic blood pressure (mmHg), mean ± SD (extremes)	97.98 ± 17.83 (60–168)	–
Heart rate (beats/min), mean ± SD (extremes)	85.76 ± 18.8 (45–157)	–
Weight (kg), mean ± SD (extremes)	73.98 ± 16.9 (42–137)	–
Height (m), mean ± SD (extremes)	1.64 ± 0.18 (1.63–1.90)	–
BMI (kg/m^2^), mean ± SD (extremes)	28.97 ± 12.66 (17.26–105)	–
Abdominal perimeter (cm), mean ± SD (extremes)	92.65 ± 13 (64–131)	–
Headache	45	23.19
Vertigo	19	9.79
Tinnitus	13	6.70
Phosphenes	10	5.15
Epistaxis	3	1.54
Palpitations	17	8.76
Chest pain	8	4.12
Dyspnoea	16	8.24
Normal ECG	76	39.17
Abnormal ECG	118	60.82
LVH	41	21.13
LAH	24	12.37
Branch blocks (RBBB, LBBB, LAHB)	18	9.27
AVB	7	3.60
Repolarisation disorders	16	8.24
Rhythm disorders (AES, VES, AF, AT)	12	6.18
Chest x-ray	–	–
CTI, mean ± SD (extremes)	0.52 ± 0.03 (0.47–0.60)	–
Cardiomegaly	12	6.18
Normal echocardiography	173	89.2

SD, standard deviation; CVRF, cardiovascular risk factors; ECG, electrocardiogram; LVH, left ventricular hypertrophy; LAH, left atrial hypertrophy; BMI, body mass index; RBBB, right bundle branch block; LBBB, left bundle branch block; LAHB, left anterior hemiblock; AES, atrial extrasystoles; VES, ventricular extrasystoles; AF, atrial fibrillation; AT, atrial tachycardia; AVB, auriculo-ventricular block; CTI, cardio-thoracic index.

The electrocardiogram was abnormal in 60.82% of cases, and the most common electrical abnormalities were left ventricular hypertrophy and left atrial hypertrophy in 21.13% and 12.37% of cases respectively.

### Therapeutic aspects

First-line treatment consisted of dual antihypertensive therapy (*n* = 133; 68.55%) in fixed dose combination (*n* = 152; 78.35%). The therapeutic classes of antihypertensive agents most commonly used were the combination of Angiotensin-converting enzyme (ACE) inhibitors (ACE inhibitors) and calcium channel blockers (30.41%), followed by the combination of ACE inhibitors and diuretics (18%). Adherence was good in 37.62% of cases. The initial treatment was modified for optimization in 32.98% of cases (Table 2).

**Table 2:** Therapeutic aspects.

Variables	*N* = 194	Percentages (%)
**Therapeutic classes**		
Angiotensin-converting enzyme inhibitors	9	4.6
Calcium channel blocker	18	9.3
Central Antihypertensive Drugs	2	1.0
Angiotensin II Receptor Blockers	3	1.5
Diuretic + Angiotensin-converting enzyme inhibitors	35	18.0
Diuretic + Angiotensin II Receptor Blockers	12	6.2
Beta-blockers + Calcium channel blocker	1	0.5
Angiotensin-converting enzyme inhibitors + Calcium channel blocker	59	30.41
Angiotensin II Receptor Blockers + Calcium channel blocker	13	6.7
Calcium channel blocker + Diuretic	11	5.7
Diuretic + Beta-blockers	2	1
Angiotensin II Receptor Blockers + Calcium channel blocker + Diuretic	9	4.6
Angiotensin-converting enzyme inhibitors + Calcium channel blocker + Diuretic	10	5.2
Angiotensin-converting enzyme inhibitors + Calcium channel blocker + Diuretic + Beta-blockers	2	1.0
**Treatment strategy**		
Monotherapy	32	16.49
Dual therapy	133	68.55
Triple therapy	27	13.91
Quadritherapy	2	1
Fixed combination	152	78.35
Therapeutic adherence	73	37.6
Normalization of BP	49	25.25

### Factors predicting normalization of blood pressure

**Figure 1:**
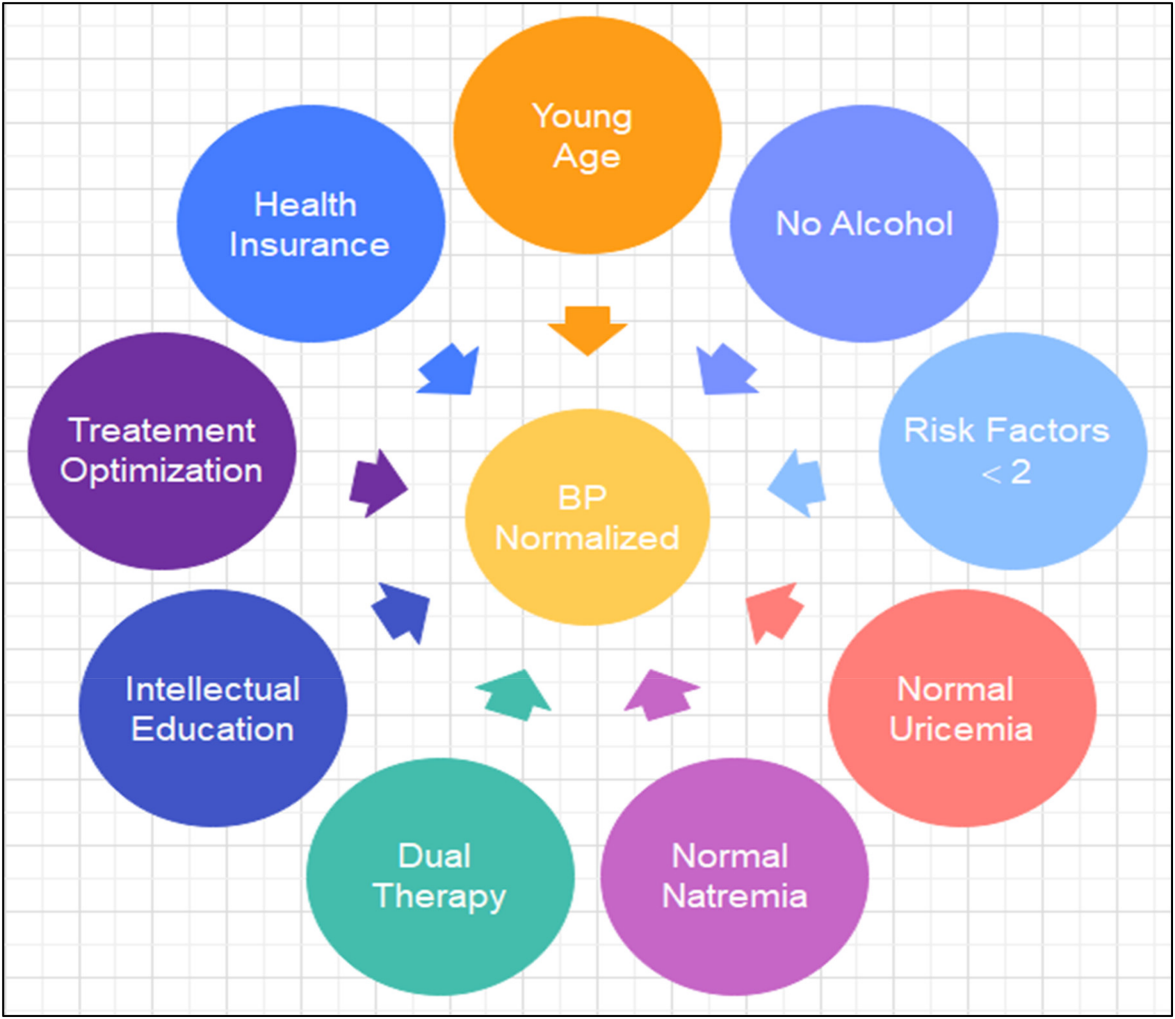
Illustrating Factors of Blood Pressure (BP) Normalization in Hypertensive Patients after short-term follow-up.

Only 25.25% of patients achieved normalization of blood pressure within 6 months, with therapeutic adherence estimated at 37.62%. About thirty-three percent (33%) of patients had their initial antihypertensive treatment modified for optimization (Figure 1).

In univariate analysis, age (*p* = 0.021), absence of obesity (*p* = 0.049), alcoholism (*p* = 0.009), number of cardiovascular risk factors <2 (*p* = 0.013), normalization of uricemia (*p* = 0.041), natraemia (*p* = 0.014), fixed dose combination therapy (*p* = 0.004), dual anti-hypertensive therapy (*p* = 0, 031), modification of treatment for optimization (*p* = 0.002), health insurance (*p* < 0.001), good level of intellectual education (*p* < 0.001) and anti-hypertensive treatment adherence (*p* = 0.004) were the main factors predicting normalization of blood pressure (Table 3).

**Table 3:** Predictive factors for normalization of blood pressure figures in univariate analysis.

Variables	BP normalized *n* = 49	BP not normalized *n* = 145	*p*-value
Age, mean ± SD (years)	55.47 ± 12.64	60.31 ± 12.54	0.021
Sex F, *n* (%)	32 (65.3)	80 (55.2)	0.437
Type II diabetes, *n* (%)	1 (2)	13 (9)	0.197
Absence of alcoholism, *n* (%)	6 (12.2)	3 (2.1)	0.009
Therapeutic adherence *n* (%)	28 (57.1)	45 (31)	0.004
Amendment *n* (%)	7 (14.3)	57 (39.3%)	0.002
Fixed therapy *n* (%)	5 (83.3)	35 (76.1)	0.004
Monotherapy *n* (%)	8 (16.3)	23 (15.9)	0.549
Fixed dual therapy *n* (%)	39 (79.6)	93 (64.1)	0.031
Obesity, mean ± SD (BMI kg/m^2^)	26.71 ± 8.6	29.61 ± 5.9	0.049
Number of CVRF, mean ± SD	2 ± 0.9	2.47 ± 1.04	0.013
Hypertension symptoms (Headache, Vertigo, Tinnitus, Phosphenes, Epistaxis)	4 (15.4)	22 (8.6)	0.239
Uric acid, mean ± SD (mg/L)	44.66 ± 10.78	56.02 ± 15.41	0.041
Natremia, mean ± SD (mEq/L)	135.71 ± 2.63	142 + 62 ± 4.82	0.014
Health insurance, *n* (%)	32 (65.3)	20 (13.8)	<0.001
Intellectual education, *n* (%)	42 (85.71)	49 (33.79)	<0.001

F, female; BMI, body mass index; CVRF, cardiovascular risk factors; SD, standard deviation, BP, blood pression.

In multivariate analysis adjusted for anti-hypertensive treatment adherence, age (OR = 1.03; 95% CI = 1.002–1.059; *p* = 0.039), absence of alcoholism (OR = 9.48; 95% CI = 2.13–42.11; *p* = 0.003), number of cardiovascular risk factors < 2 (OR = 1.52; 95% CI = 1.06–2.16; *p* = 0.021), normalization of uricemia (OR = 1.05; 95% CI = 1.00–1.11; *p* = 0.039), natraemia (OR = 1.01; 95% CI = 1.00–1.03; *p* = 0.021), dual therapy (OR = 0.40; 95% CI = 0.18–0.90; *p* = 0.027), modification of treatment (OR = 4.22; 95% CI = 1.71–10.37; *p* = 0.002), good level of intellectual education (OR = 10.40; 95% CI = 4.31–25.10; *p* < 0.001) and health insurance (OR = 0.09; 95% CI = 0.04–0.21; *p* < 0.001) were the main factors predicting normalization of blood pressure (Table 4).

**Table 4:** Predictive factors for normalization of blood pressure figures in multivariate analysis adjusted for therapeutic compliance.

Variables	Odds ratio	95% CI	*p*-value
Young age	1.03	1.002–1.059	0.039
Absence of alcoholism	9.48	2.13–42.11	0.003
Low number of CVRF (<2)	1.52	1.06–2.16	0.021
Normalization of uricemia	1.05	1.00–1.11	0.039
Natremia normalization	1.01	1.00–1.03	0.021
Health Insurance	0.09	0.04–0.21	<0.001
Intellectual education	10.40	4.31–25.10	<0.001
Bitherapy	0.40	0.18–0.90	0.027
Change in treatment for optimization	4.22	1.71–10.37	0.002

CVRF, cardiovascular risk factors.

## Discussion

### Normalization rate of blood pressure and ideal blood pressure target

Recent American and European guidelines on the therapeutic strategy for hypertension recommend a significant reduction in blood pressure (BP) to below 130/80 mmHg as the blood pressure target for most hypertensive patients on treatment (10–13, 16, 20–22). Initially, the first blood pressure target when treating patients with antihypertensive drugs should be a blood pressure below 140/90 mmHg (6, 22). Secondly, an optimal BP of less than 130/80 mmHg could be aimed for (10, 16, 23). However, caution should be the rule, as lowering blood pressure too severely to below 120/70 mmHg may be harmful to the hypertensive patient, exposing him or her to systemic organ hypoperfusion (10). In our series, out of 194 hypertensive patients, only 49 patients (25.3%) achieved normalization of blood pressure within 6 months of starting anti-hypertensive treatment. Clara K et al, in 2013, in Canada, in a cross-sectional study of a multinational, multicentre population, found a rate of BP control varying between 20.7% and 40% in treated hypertensives. The lowest rates of BP normalization were found in developing countries in Africa, and the highest rates in developed countries and South Africa (3). These rates are lower than those of Lucinda Calas et al. in Mayotte (24) in 2019, Al-Saadi R in Greece in 2011 (25), Donna Shelley in the USA in 2011 (26) and Onwukwe in South Africa in 2012 (27), who respectively found a control rate of 30.2%, 55.6%, 49.8% and 57% in hypertensive patients on pharmacological treatment (4).

### Predictive factors for normalization of blood pressure

The factors involved in normalizing BP are numerous and vary according to the authors. Our data are similar to those in the literature, which found that good control of blood pressure was associated with young age, absence of obesity and alcohol consumption, low number of cardiovascular risk factors, correction of uricemia and natremia, use of anti-hypertensive therapies in fixed combinations, modification of treatment, health insurance, adherence with anti-hypertensive treatment and a good level of intellectual education (3, 9, 28–32). In our study, normalized blood pressure was significantly associated with a younger age, less than 60 years. Sheleme T et al. in 2022 (1) made the same observation and explained this phenomenon by the fact that comorbidities increase with ageing and do not facilitate the taking of medication. These results contrast with those of Teshome et al. in Ethiopia (28) in 2018, who found better control of blood pressure in hypertensive patients aged over 60 compared with younger subjects. This could be explained in part by young people's refusal to accept the disease and subsequent therapy. Correction of hyperuricaemia was a predictive factor for normalization of blood pressure in treated hypertensive patients. These results have been corroborated by other authors such as Borghi et al. (33) in 2022 in Italy. As in our series, non-adherence to anti-hypertensive treatment has been recognized as a major problem associated with uncontrolled severe arterial hypertension and is thought to be responsible for approximately 50% of therapeutic failures of anti-hypertensive drugs (9, 34–36). Currently, learned societies (16) strongly recommend the use of a fixed combination of dual therapy during initial treatment for most patients in order to achieve greater efficacy, good adherence with treatment and more rapid attainment of the ideal blood pressure target. Almost 65% of our patients with normalized blood pressure were covered by health insurance. Similar studies have reported the absence of health insurance as a factor associated with poor blood pressure control (9, 37). As reported by other authors (9), our results demonstrate that blood pressure control requires a good level of intellectual literacy on the part of the patient.

### Reasons of nonadherence to antihypertensive treatment

Adherence is defined as the extent to which the patient follows prescriptions or oral recommendations from a health care provider (16). It is reported that only approximately one-fourth (25%) are adherent to antihypertensive treatment (38–40). In our series, 37.62% of patients were adherent. The reasons for this low adherence rate in our series overlap with those mentioned by the WHO (41), namely: problems with drug reimbursements, side-effects, complexity of drug regime or interference with daily routine, lack of symptoms or presence of comorbidities with a long list of medications to ingest, low self-efficacy or inaccurate beliefs about medications, poverty with high cost of medications, lack of family support or unemployment. The generally asymptomatic nature of hypertension is likely to increase the risk of this nonadherence.

## Strenghs and potential limitations of our study

This analytical study allowed us to clearly predicting Factors of Blood Pressure Normalization and its methodology was based on recent Hypertension management guidelines. However it was a single-center retrospective study with possible lack of representativity of patients group; sport and eating habits were not evaluated. The indirect method (Self report) we used to measure the medication adherence was simple, easy, inexpensive method, integrated into the care relationship. However, it is known to presente a low reliability, an overestimation of adherence and a risk of focusing the relationship on adherence (19, 42). Also, the lack of quantitative data on the reasons why patients were non-adherent is a limitation.

## Perspectives

Only 25.25% of our patients achieved normalization of blood pressure. These results are consistent with regional data. Indeed, since 2017, several studies have confirmed suboptimal blood pressure control in sub-Saharan Africa countries (9%–47.3%) (43–45).

The normalization of blood pressure is a major challenge for the practitioner. It is a health emergency which requires the implementation as a priority of a national health program aimed at has:

• Promote 100% comprehensive health insurance, allowing our patients to have health security and improve therapeutic compliance.
• Promote literacy courses to improve the educational level of our populations.
• Promote therapeutic education and strict compliance with the health and diet regime with priority given to the low-sodium diet.
• Make antihypertensive treatment available at lower cost and accessibility for all our populations.
• Develop a system of continuing medical training with a view to updating practitioners' knowledge.

## Conclusion

The rate of blood pressure normalization remains poor worldwide and even more worrying in our context. Control of cardiovascular risk factors, fixed dual therapy, adherence with treatment, appropriate modification of antihypertensive treatment, normal uraemia and natraemia, health insurance and an intellectual education are the main factors in normalizing blood pressure levels.

## Data Availability

The raw data supporting the conclusions of this article will be made available by the authors, without undue reservation.
